# Aerial tactile perching via an anthropomorphic hand with embodied soft tactile receptors

**DOI:** 10.1038/s44182-026-00109-9

**Published:** 2026-08-22

**Authors:** Anton Bredenbeck, Anish Jadoenathmisier, Salua Hamaza

**Affiliations:** BioMorphic Intelligence Lab, Department of Control & Operations, Aerospace Engineering, TU Delft, The Netherlands

**Keywords:** Engineering, Mathematics and computing

## Abstract

Aerial robots are widely employed for exploration, inspection, and environmental monitoring, where their agility and maneuverability are strong assets. However, their endurance remains limited, inhibiting their applicability in long-term missions. Perching, the ability to attach to environmental structures and rest with minimal power, offers a solution. Yet existing methods typically rely on bespoke attachment mechanisms tuned to a single, known, and predefined target, as well as vision-based target detection systems, prone to noise and occlusions, forcing reliance on brittle feed-forward control. We introduce a tactile-driven perching strategy for aerial robots that refines pose through touch. The system integrates a compliant anthropomorphic hand with embedded binary tactile sensors, enabling closed-loop alignment and grasp stability assessment through direct physical interaction. In simulation, the method achieves over 99% perching success across diverse geometries and pose errors up to 0.6 m and 50°. Hardware experiments validate robust perching across 26 real-world trials on diverse structures, despite corrupted pose estimates. By embedding tactile feedback into perching, this work advances a new paradigm, enabling micro aerial vehicles to exploit contact as informative feedback rather than relying solely on pre-contact visual estimates, facilitating robust autonomous perching on diverse, previously unseen targets in unstructured environments.

## Introduction

MAVs have become increasingly essential for applications in exploration, surveillance, and environmental monitoring. Their agility and ability to operate in confined or cluttered environments make them particularly suited for urban missions^1^, time-critical search-and-rescue deployments^2^, and ecological data collection^3–5^. Nevertheless, their performance is fundamentally limited by poor flight endurance: even under ideal conditions, most MAVs can travel only a few kilometres, or sustain flight for tens of minutes^6^. This constraint severely restricts their ability to perform long-duration tasks, such as environmental monitoring over extended time horizons.

Perching refers to the behavior of biological systems—typically birds or arboreal animals—to rest on a surface by maintaining contact at one or more attachment points. In aerial robotics, perching is often exploited in the context of drones to extend mission duration while maintaining an energy-free resting state. A wide spectrum of perching technologies has been explored in aerial robotics. Design-based solutions typically employ robotic grippers mounted underneath the vehicle, such as avian-inspired claws^7,8^, bistable elements^3^, or passive latching systems for fixed-wing drones^9^. The main drawback of design-based solutions is, however, their reliance on absolute knowledge of the target location and accurate position tracking during the approach phase. These strong assumptions enable the flying gripper to align with the target; however, with little to no tolerance for uncertainty in the target pose estimate.

Besides these fundamental limitations, there are also alternative perching methods such as adhesive^10,11^, suction-based^12,13^, and magnetic^14,15^ mechanisms, which allow surface attachment but impose strict constraints on the surface cleanliness, smoothness, or material composition for perching. Other design-based strategies leverage friction or geometric interlocking: frictional perching for canopy structures^16^, hook-based attachment for rough surfaces^17^, custom grippers for well-defined geometries^18–20^, and lightweight tensile perching designs^21, 22^.

For problems of the broader class of aerial interaction, prior works have closed the loop with onboard vision: compliant grippers paired with learned target detectors have been demonstrated for aerial grasping^23–25^, and stereo and RGB-D pipelines for aerial perching^26^. Across both tasks, these vision-based pipelines share well-known failure modes: they are trained on a specific set of objects, require an unoccluded view, and most crucially lose the target whenever it leaves the camera’s field of view or is occluded by the gripper itself during the approach. The grasping works do, however, demonstrate two enduring advantages of compliant grippers for aerial physical interaction: shape-adaptive grasping across a range of target geometries, and absorption of impact forces transmitted to the platform base. However, in all proposed designs, these grippers require sustained actuation to maintain a closed grasp, making them energy inefficient for perching. Furthermore, they also provide no feedback on contact establishment or grasp quality, leaving the controller blind to the moment of capture and to any post-grasp slip.

Similar lessons about compliance, underactuation, and feedback have been distilled in conventional ground-based manipulation, where the design space of anthropomorphic and underactuated hands is mature and is the subject of systematic survey^27^. Tendon-driven anthropomorphic hands exploit synergies (low-dimensional joint-space couplings) to produce diverse grasp modalities (e.g., power and precision) from a minimal actuator set, conforming to object geometry without measuring or explicitly modeling the target^28^. Across this space, underactuated tendon-driven architectures stand out for delivering anthropomorphic dexterity and inherent mechanical compliance from a minimal motor count, as exemplified by postural-synergy designs derived from human grasping data^29^, adaptive-synergy designs with antagonistic tendons and tactile fingertips^30^, and design-time parameter optimization of underactuated hands against analytical grasp-quality metrics^31^. Commercial platforms such as the 980 g BarrettHand^32^ showcase the industrial usage of underactuated grasping. However, while achieving impressive dexterity with minimal actuation, their mass envelope still disqualifies them from use in aerial robotics and, as in the previous examples of aerial grippers, their dexterity requires actuation to maintain a closed grasp.

Task-space feedback for manipulation in the form of distributed tactile measurements has likewise been extensively studied^33^. On one side, tactile and joint-angle signals alone have been shown sufficient for blind grasp-stability classification, without any visual or geometric prior^34^; on the other, distributed tactile feedback can serve as an active control signal to generate whole-hand envelope grasps on unknown targets^35^. Many of these demonstrations, however, are realized on heavy, ground-fixed manipulators, with high-fidelity tactile sensors while closing the tactile loop to the actuated fingers rather than using minimal tactile sensors that inform the control actions of the base.

Despite substantial progress in perching mechanisms for drones, deploying these methods in unstructured environments remains challenging. All grippers still require extremely precise maneuvers to guarantee alignment, which are impractical in cluttered settings or in the real world. Adhesive, suction, magnetic, and geometry-specific methods further constrain the range of surfaces suitable for perching. Moreover, nearly all existing approaches rely on accurate prior knowledge of the target’s position and orientation. In the studies mentioned, aerial perching is typically conducted in laboratory settings with Motion-Capture systems providing the pose of the perching target beforehand, and the actual maneuver is then executed in an open-loop fashion with a precise estimate of the target pose, tracking a predefined waypoint trajectory. These challenges highlight the need for additional perception modalities to support aerial perching maneuvers, beyond visual guidance. In this context, continuous tactile feedback can more effectively guide the maneuver, enable real-time alignments during grasping to adapt to diverse target geometries, and validate the robustness of the grasp during interaction. Biological systems embody this principle seamlessly: perching animals rely on tactile feedback and bodily compliance when engaging with their surroundings, using touch to adjust posture and grasp to the environment. Inspired by these behaviors, tactile sensing in aerial robotics has emerged as a novel tool to provide continuous feedback in aerial physical interaction, directly in the task-space^36–40^.

Building on this insight, we propose an aerial tactile perching framework that equips a compliant anthropomorphic hand with soft binary tactile sensors on an aerial platform. Starting from only a rough estimation of the target location in space, the system continuously leverages tactile feedback to infer the precise location and orientation of the perching structure, to guide real-time flight adjustments during the approach, and to evaluate grasp stability upon perching. By closing the feedback loop directly in the task space, our novel approach enables robust, vision-free aerial perching that accommodates substantial misalignments to the perching target and unpredictable surface geometries. The main contributions of this work are:

- A compliant anthropomorphic hand equipped with soft tactile pads that act as sensorized phalanges, providing both environmental perception and passive adaptation to diverse structures.

- An aerial tactile perching framework that continuously refines the MAV pose during the perching maneuver using embodied tactile feedback.

- A tactile-based grasp validation strategy that ensures secure attachment before finalizing the perch, enabling reliable, energy-free operation.

## Results

### Anthropomorphic hand with distributed touch

**Fig. 1:**
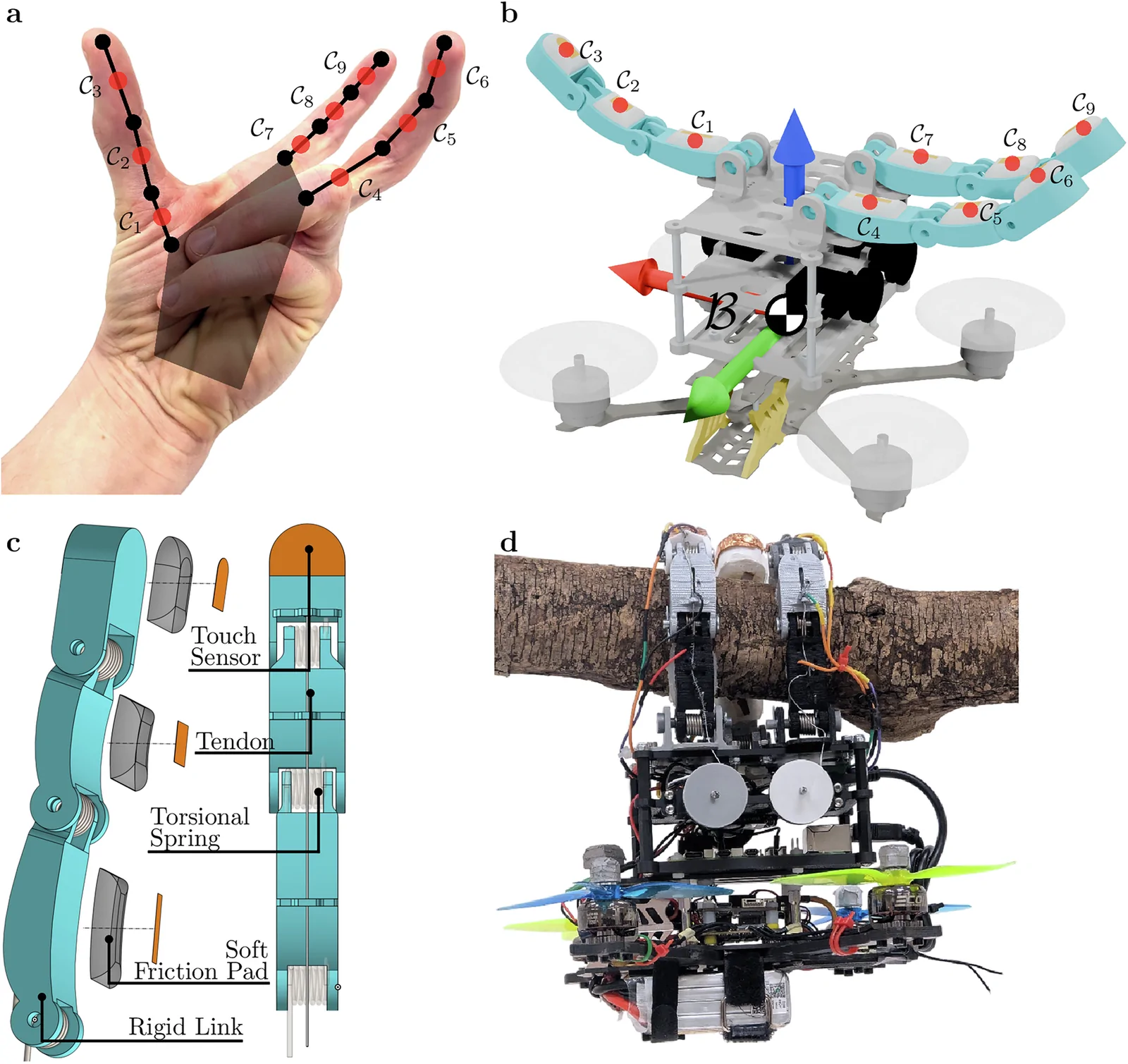
Biological inspiration, design, and implementation of the tactile flying gripper. **a** The human hand leverages fingers in an antagonistic configuration relative to the thumb (left), which enables grasping objects of varying geometries and sizes while providing tactile feedback along multiple planes and directions. **b** Our proposed anthropomorphic hand adopts a similar configuration with distributed touch sensing. Nine tactile sensors (${{\mathcal{C}}}_{1}$ to ${{\mathcal{C}}}_{9}$) are positioned at the center of each phalange, providing a binary contact signal whenever a sensor ${{\mathcal{C}}}_{i}$ contacts the environment. The drone body frame of reference, ${\mathcal{B}}$, is located at the CoM. **c** The mechanical implementation of the tactile hand is mounted on top of a 4-inch quad frame. We highlight the individual components. Capacitive touch sensor: copper foil connected to a capacitive sensing board provides binary information about touch events. Note that the capacitive sensor on the last phalanx is connected to copper foil on the front and the back of the phalanx, allowing for two-sided contact detection. Motor and tendon tension spool: by tensioning the tendon, the finger opens while the torsional springs in the joints ensure passive closing. All components are operated by a RaspberryPi 5 companion computer and powered by a 4S battery.

Designing an anthropomorphic tactile hand for aerial robots begins with understanding how human fingers achieve reliable grasping through synergistic morphology and tactile perception. Translating this biological inspiration to drone applications, moreover, requires reconciling the design with the strict payload and mass limitations of micro aerial vehicles (MAVs). Drones, in fact, rely on small batteries to power both onboard electronics and propulsion, where propulsion alone consumes nearly all the available energy. As a result, even small increases in payload can substantially reduce flight time and negatively impact maneuverability. This makes simplicity, low mass, and minimal power consumption essential design requirements for any tactile hand intended for aerial manipulation. Furthermore, such an anthropomorphic hand should reliably sustain the MAV’s weight while requiring minimal actuation or power, and adding minimal mass to the system. With these constraints in mind, we arrive at the design depicted in Fig. 1. Each finger in our system is composed of three rounded phalanges connected by revolute joints that mimic the human finger’s kinematics. Torsional springs at each joint provide passive stiffness and set the hand’s nominal posture to a naturally closed state without requiring continuous actuation. The phalanges incorporate a soft silicone interior that supplies friction and compliance for interacting with objects of varying shape, geometry, and texture, mimicking the morphologies of biological finger pads. The phalanges follow the human anatomical pattern in which the proximal segment is longest, followed by the middle and then the distal. This configuration evolved in primates to optimize grasping across diverse objects^41^, in particular for suspensory and climbing behaviors. By replicating this morphology, we aim to leverage these evolutionary advantages to improve the hand’s adaptability to various perching targets. To convey the sense of touch in a distributed manner, tactile sensors based on capacitance are mounted on the surface of each phalanx and provide binary contact information spread across a soft, compliant surface. A single artificial tendon running along the back of each finger connects the phalanges to a lightweight actuation spool: tightening the tendon opens the finger, while releasing it allows the springs to close the finger passively. This combination of compliant morphology, passive mechanics, and minimal actuation results in a tactile hand that is both energetically suitable for aerial perching applications and adaptable to diverse target geometries.

### Tactile perching control

**Fig. 2:**
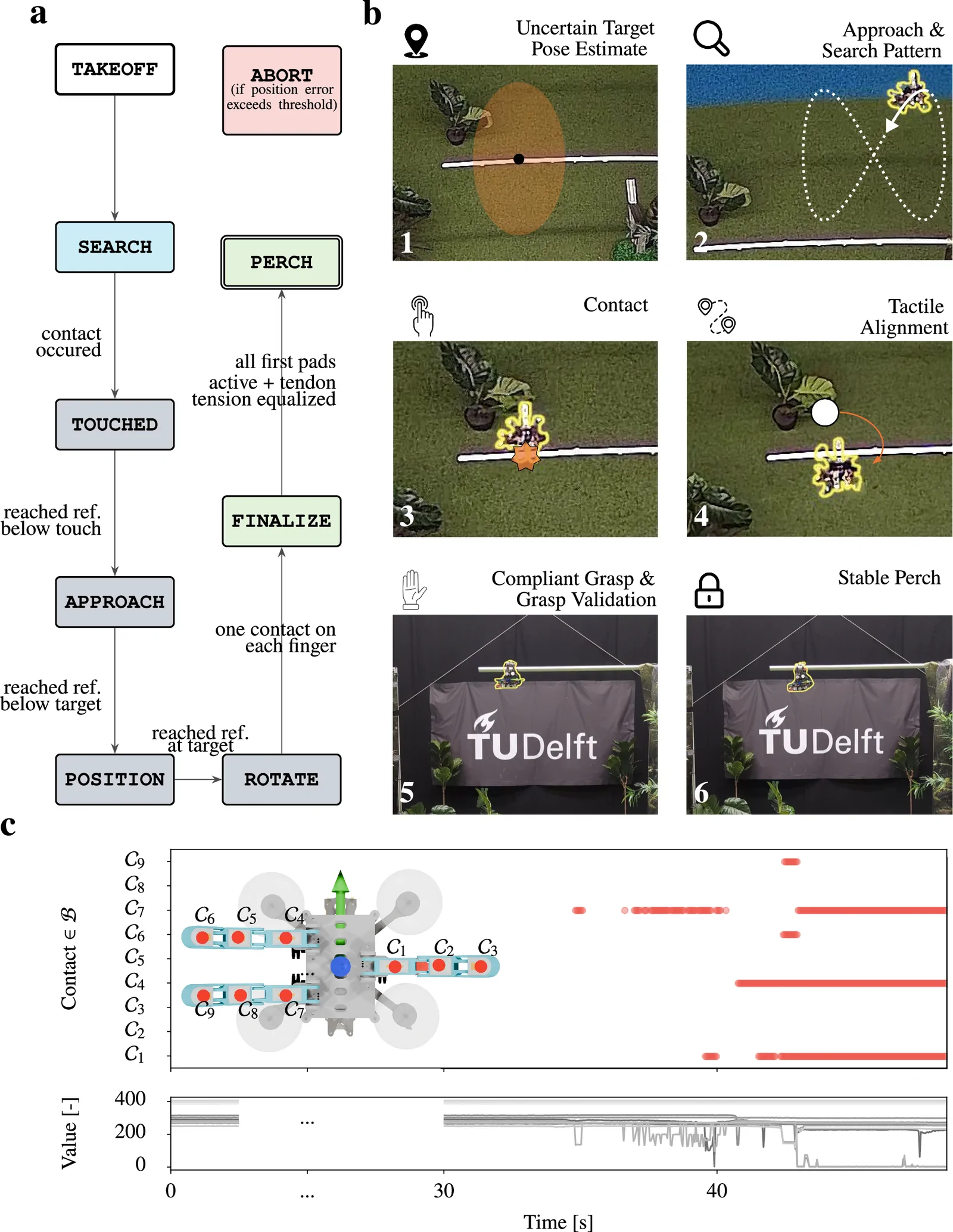
State machine, resulting procedure, and signal processing for the tactile perching approach. **a** A block diagram of the state machine used to implement the tactile perching approach. **b** The resulting behavior of the physical prototype when commanded by the state machine. The system transitions between TAKEOFF, SEARCHING, TOUCHED, APPROACH, POSITION, ROTATION, FINALIZE, PERCH, and ABORT, driven by contact events and pose convergence. **c** Processing of the raw capacitive values from the touch sensors to binary contact signals. An example trial (Trial XVII in Fig. 4) showcases the functionality of the capacitive touch sensors. By thresholding the difference from the nominal value, we obtain a robust binary signal.

As observed in nature, touch is a critical sensing modality to align the airborne agents with their perching target. By reacting to tactile cues, the MAV can adjust its position and alignment to ensure a safe grasp, robust to position and alignment offsets. This work uses a Finite State Machine (FSM), as depicted in Fig. 2, that progresses through various states to bring the MAV from a (potentially incorrect) initial target pose estimate to a safely perched state. Hereby, the FSM relies on the following assumptions about the environment and the perching target:

**A.1** The initial target pose estimate is in the vicinity of the true target pose.

**A.2** The target object has a characteristic diameter that fits within the gripper.

**A.3** The area surrounding the initial target estimate is free of obstacles, other than the target itself.

The following section will introduce the FSM states {TAKEOFF, SEARCH, TOUCHED, APPROACH, POSITION, ROTATE, FINALIZE, PERCHED, ABORT} and their transitions.

In TAKEOFF, the MAV executes a takeoff from the ground. It will approach a pre-defined takeoff location above the origin. Once the position error satisfies ∥**e**_pos_∥ < *ϵ*_pos_, the FSM transitions to SEARCH.

In SEARCH, the MAV follows a search pattern defined by a vector field ${\bf{g}}({{\bf{p}}}_{{\mathcal{B}}},{{\bf{p}}}_{{\mathcal{T}},0},t)$, following the implementation in ref. ^37^. Additionally, the search pattern commands a hand opening vector ${\bf{s}}({{\bf{p}}}_{{\mathcal{B}}},{{\bf{p}}}_{{\mathcal{T}},0},t)$, with one value in [0, 1] per finger, denoting the degree of opening of the finger. Hereby ${{\bf{p}}}_{{\mathcal{B}}}$ is the current position of the MAV in the world frame, ${{\bf{p}}}_{{\mathcal{T}},0}$ is the initial pose estimate in the world frame, and *t* is the current time. Hereby, the vector field specifies the target velocity of the MAV at a certain location and time, and **s** is the hand opening state. We choose to implement the vector field as a height-stepping sinusoidal figure-eight pattern as depicted in Fig. 2. The search pattern is planar; after each completed cycle of the search pattern, we increase its altitude. At the same time, we also command the MAV to slowly open and close the hand such that it reaches its opening apex at the apexes of the figure-eight. This increases the reach of the search pattern and therefore the chance of a touch event occurring. A touch event triggers a transition to TOUCHED.

In TOUCHED, the MAV exploits the first contact to obtain an initial target estimate and moves to a reference position offset below and away from the contact point (toward the direction of the body frame at the center-of-mass (CoM) of the MAV ${\mathcal{B}}$) by *Δz* and *Δγ*, with the hand fully opened. When ∥**e**_pos_∥ < *ϵ*_pos_, the FSM enters APPROACH.

In APPROACH, the MAV moves to a reference position offset below the contact-based target position estimate and switches to POSITION once the position error again falls below *ϵ*_pos_.

In POSITION, the MAV moves to the target position estimate without any offset, after having positioned itself below during the previous state; satisfaction of ∥**e**_pos_∥ < *ϵ*_pos_ then leads to ROTATE.

In ROTATE, the MAV incrementally closes its fingers to provoke contact, using the pattern of contact to infer the target orientation: fingers that detect contact stop closing, and the MAV yaws away from them until the contact is no longer present. Since the closing values for all fingers increase monotonically, i.e., the fingers do not reopen during this state, the process is guaranteed to converge to a fully closed grasp. Furthermore, small lateral position corrections are applied if contact is unilateral. This process continues until all arms report at least one pad with consistent contact, at which point the FSM transitions to FINALIZE.

In FINALIZE, all fingers are driven to a fully closed configuration and the grasp is validated by checking that all bottom pads are active and tendon tensions have equalized. A valid grasp leads to the terminal PERCHED state, in which the MAV is supported by the target and its motors are turned off.

From any state, if the tracking error exceeds the safety threshold *ϵ*_abort_, the FSM transitions to ABORT. In ABORT, the MAV returns to a safe hover above the origin and re-enters SEARCH, thereby re-initializing the perching attempt.

### Robustness to target offsets

**Fig. 3:**
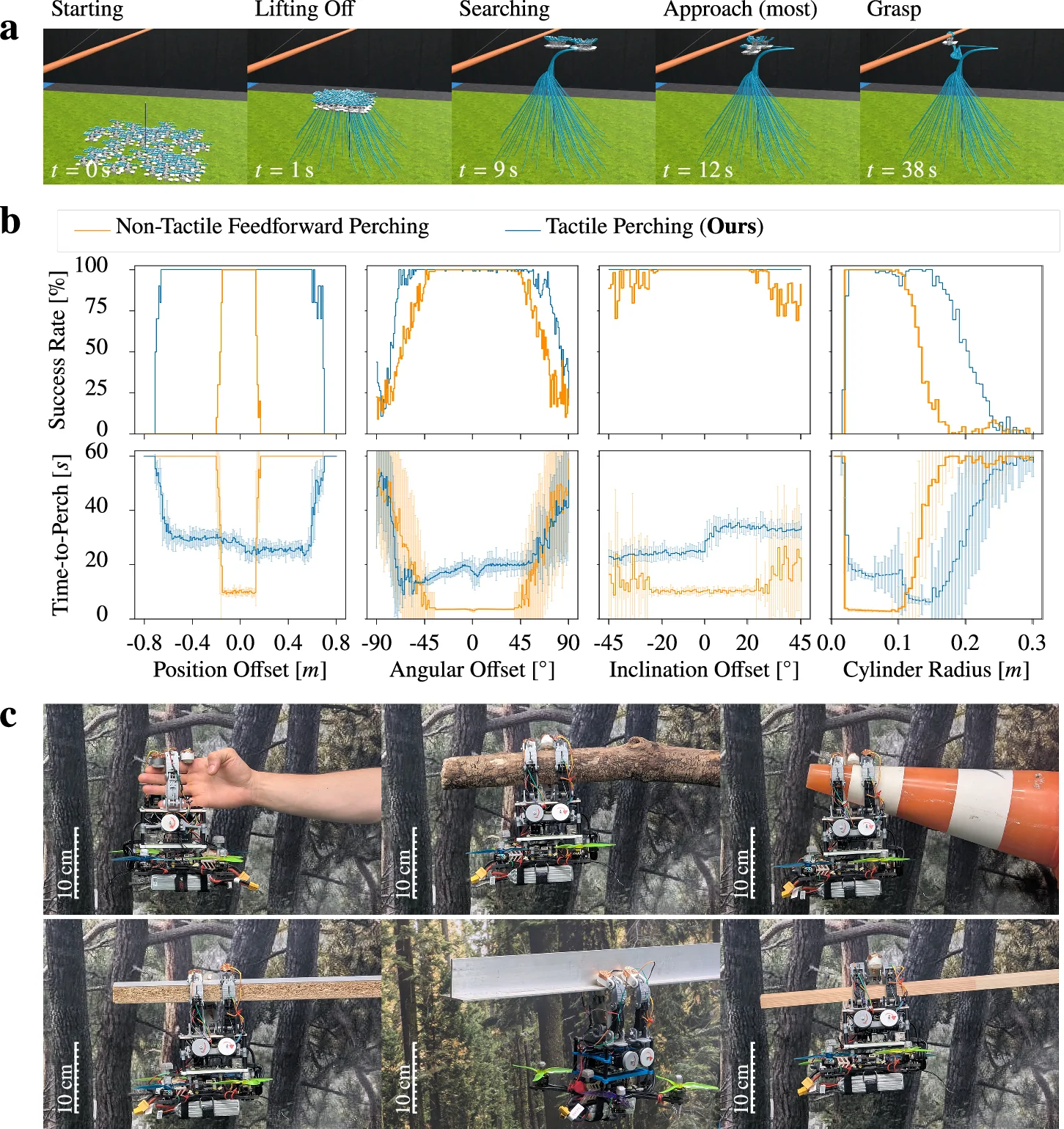
Robustness of the proposed approach to target pose estimation offsets, and target sizes. **a** Still images of 100 trials of a single experiment. **b** The success rate and the mean time to perch with its standard deviation. We compare a baseline feed-forward perching strategy (orange) with our tactile-based perching strategy (blue). (From left to right) 100 trials for various positional offsets, 100 trials for various rotational offsets, 100 trials for various inclinational offsets, and 100 trials for various target sizes. **c** The anthropomorphic, compliant hand supporting the full MAVs weight while hanging from various structures of different diameters and shapes. Thanks to its parallel revolute joints, the gripper exhibits passive compliance, allowing it to adapt to a wide range of targets. Even objects with non-uniform diameters and low-friction plastic or metallic surfaces, such as a traffic cone or a structural T-Beam, can be grasped and support the MAVs weight, as the arms close passively and compress until the contact force balances the spring torsion.

In order to quantitatively evaluate the performance of the proposed tactile-based perching strategy, we perform a Monte-Carlo simulation study. We conduct 100 simulated trials for various offsets in initial target position and orientation offsets as well as different target cylinder radii. We employ the Genesis World simulator^42^ in which a 6-degree-of-freedom (DoF) model of the MAV and a tendon-actuated gripper with linear joint stiffness is simulated. The low-level position, orientation, and rate controllers of the MAV are fed noisy state measurements to increase representativeness of real-world conditions. Figure 3 shows a set of still images of one of the experiments as well as the resulting success rates and mean time to perch for the proposed method and a baseline feed-forward perching strategy without tactile feedback. The baseline feed-forward perching strategy is implemented by commanding the MAV to approach the target at its initial pose estimate from below, and fully closing the hand after reaching the target position estimate, without considering any feedback. Supplementary Movie 1 shows animations of representative trials for rotational, inclinational, and positional misalignments, as well as a single experiment from the Monte-Carlo study. Except for the inclinational sweep, the tactile-based perching strategy outperforms the feed-forward baseline in all experiments, showing a larger bandwidth with success rates above 99% for positional offsets, for orientation offsets and for target cylinder radii. The inclinational sweep shows that the underactuated gripper alone is capable of adapting to the target orientation, enabling a robust perching strategy.

To showcase the ability of versatile grasping, we perform a series of static perching experiments with targets of different shapes and sizes and surfaces. Each finger of the gripper contains three parallel revolute joints, making it compliant to different structures, and thus enabling the MAV to attach itself to differently shaped targets. Figure 3 shows the gripper successfully hanging from various structures, including a human arm, multiple tree branches of different diameters, rectangular wooden beams, and a traffic cone with a varying diameter and a very s low-friction plastic surface. In each case, different phalanges take on the role of the main contact point, and consequently a different revolute spring carries the main portion of the load. This demonstrates the gripper’s adaptability solely through its mechanically compliant design.

**Fig. 4:**
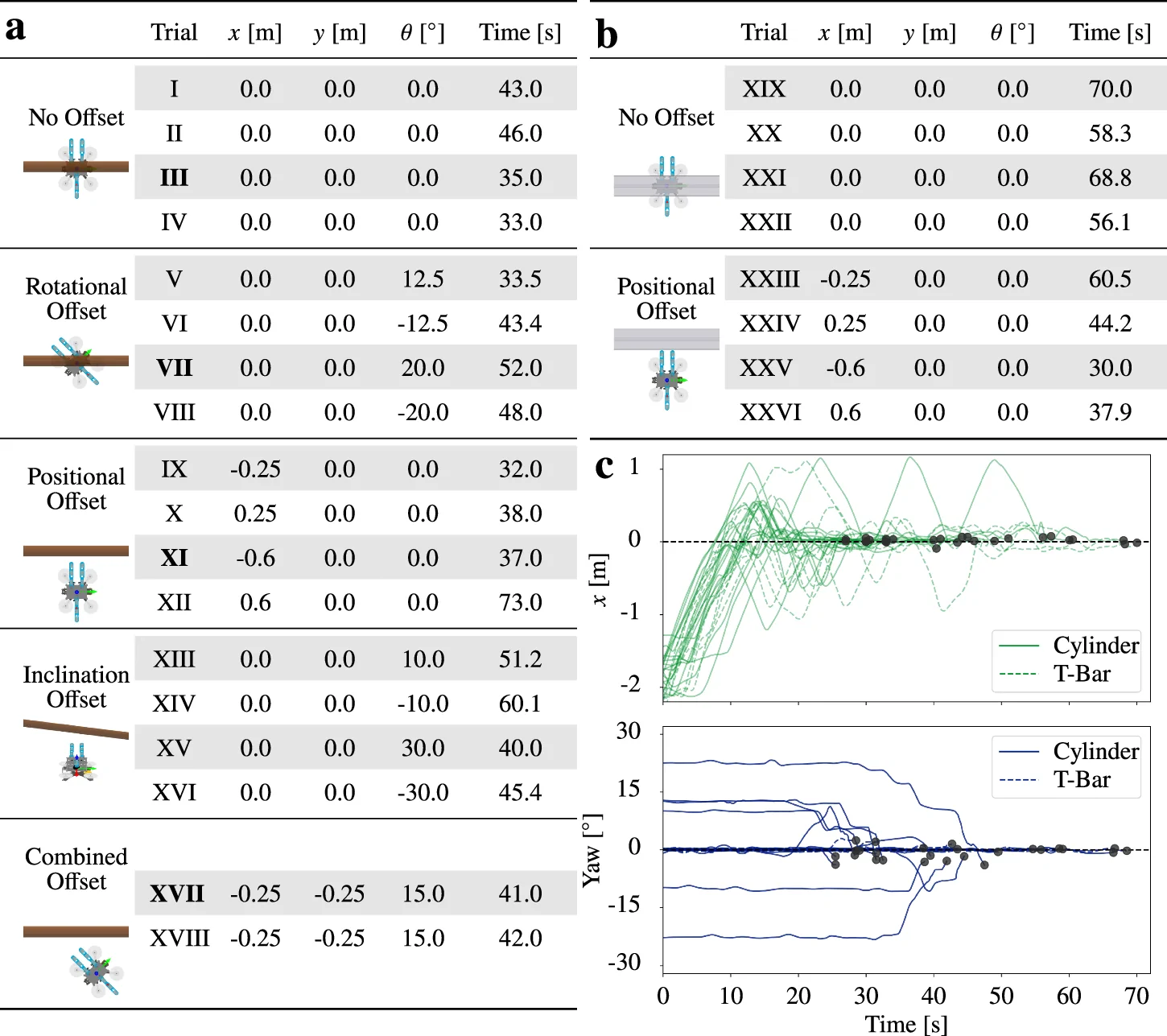
Experimental setup (offsets) and result (success & time-to-perch) of each trial with the physical prototype. **a**, **b** Table of initial target pose estimation offsets (*x* and *y* position and angle *θ* for yaw and inclination) for all trials with the cylindrical and T-Bar perching target, respectively. The trials highlighted in bold are further visualized in Fig. 5. **c** Time series data of the MAV's *x* position and yaw angle, showcasing all trials aligning with and converging to the target pose. The data are normalized to have the target pose at the origin. Note that the data is normalized such that the approach happens entirely along the *x* axis. The black dots indicate when a trial has successfully reached the perching state.

**Fig. 5:**
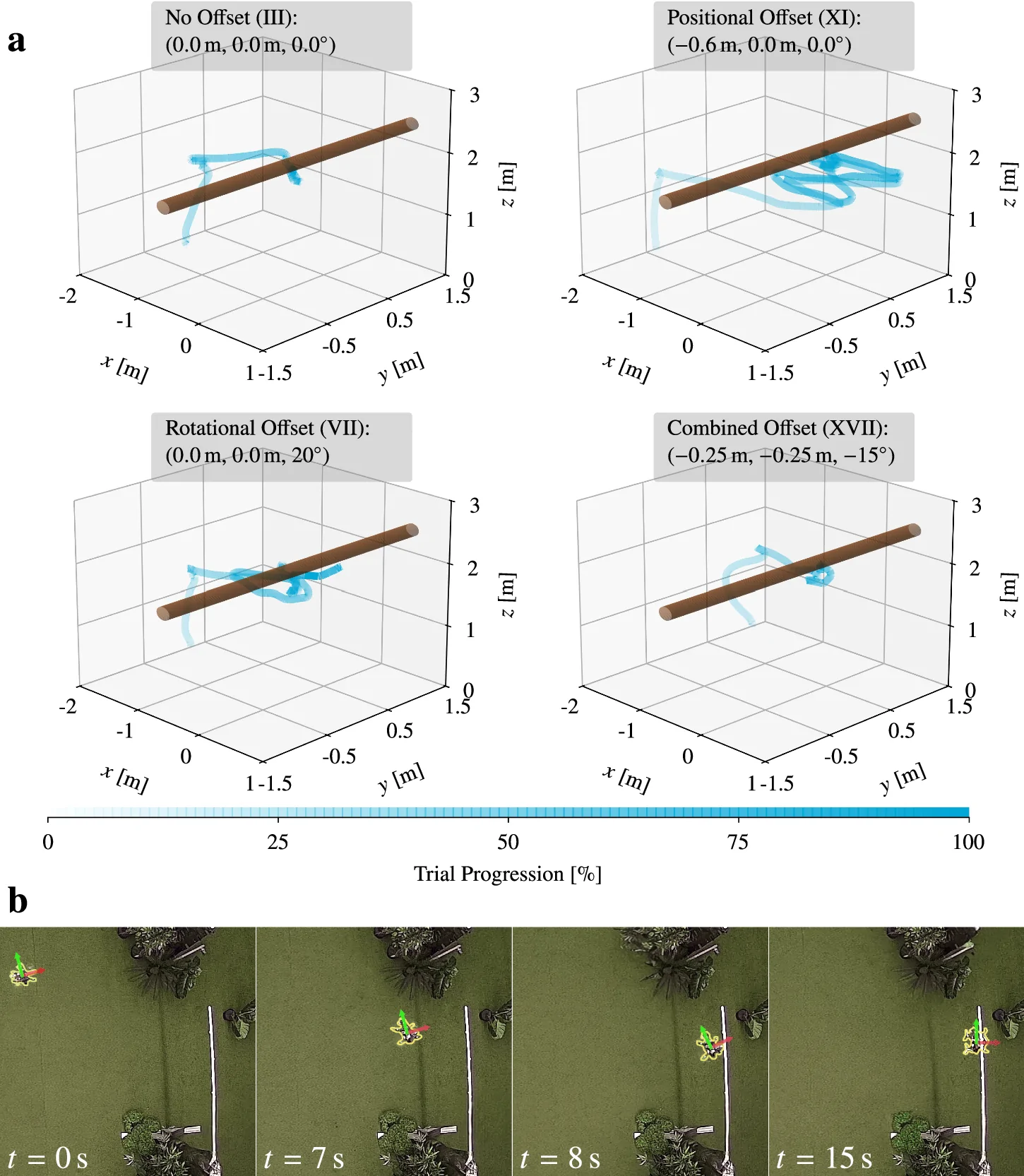
Visualizations of experiments with the physical prototype. **a** Three-dimensional trajectory plots of selected trials. For different initial takeoff positions and initial offsets, contact occurs at different times. However, in all cases the proposed approach is able to align with and successfully perch on the target. **b** Snapshots of Trial XIII from Fig. 4 showcasing the different phases of the tactile perching maneuver. See Fig. 4 for the initial offsets.

Using the physical prototype introduced before, we perform a series of flight experiments to validate the proposed tactile-based perching strategy. We conduct 26 trials with different target geometries and initial target pose estimate offsets as detailed in Fig. 4. Supplementary Movie 2 shows a close-up of the tactile perching procedure as well as a top-down view of all trials. All data collected during these experiments are provided via our repository (https://github.com/BioMorphic-Intelligence-Lab/feely_drone). Figure 5 shows still images of one of the trials as well as the time series data of the *x* coordinate and yaw angle for all 26 trials. It shows that in all trials the MAV is able to converge to the correct target position and orientation. Naturally, some trials take longer, as the duration of the searching phase is dependent on the drone starting position, the initial target position offset, and the initial target orientation offset, which lead to different contact times.

**Fig. 6:**
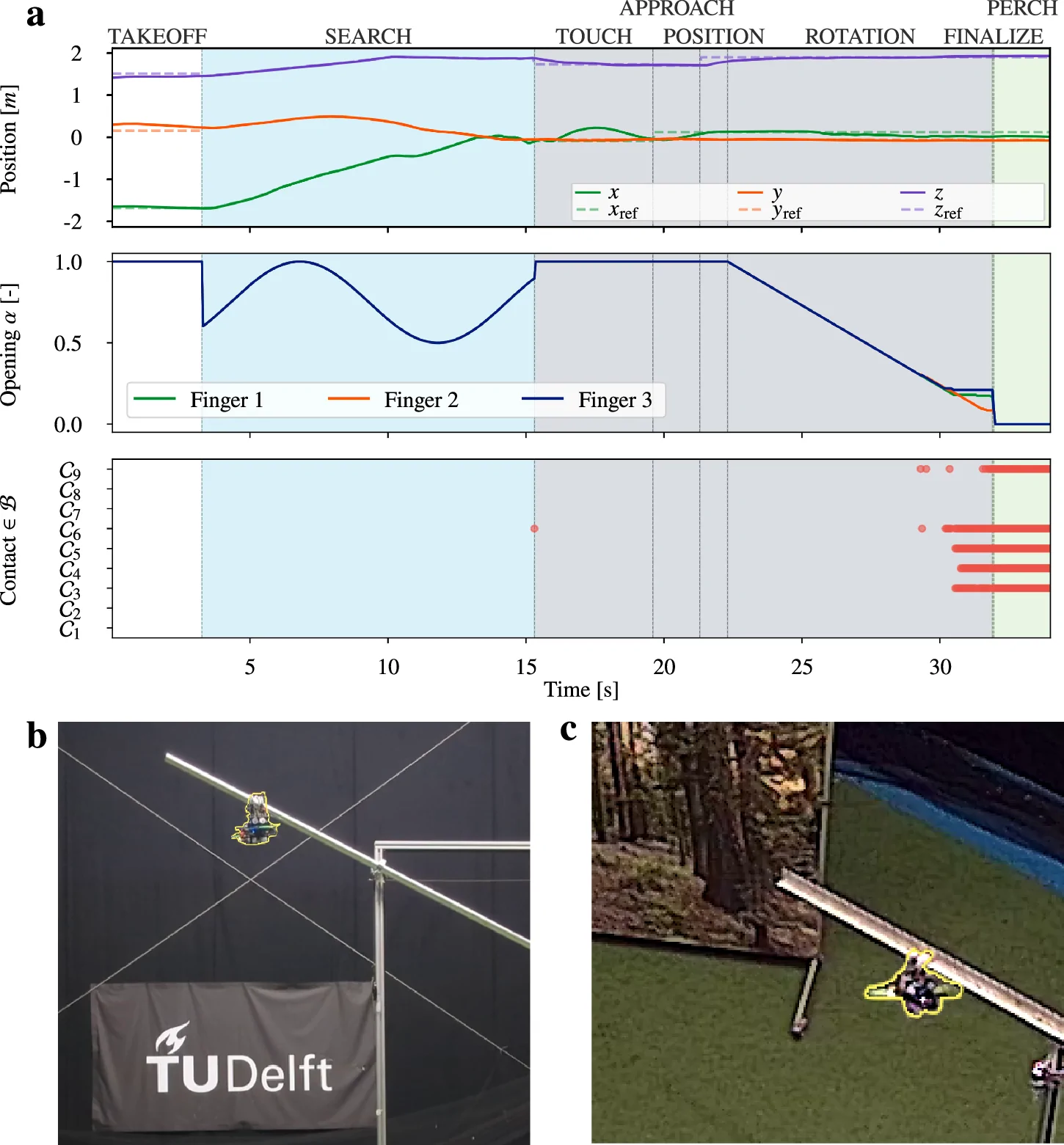
Highlights of the proposed system's capabilities. **a** Full trial overview plot for trial III (c.f. Fig. 5) showcasing the position, arm opening states, and contact signals over the different phases of the tactile perching procedure. Initially, the MAV takes off, then it proceeds to search for the target; contact occurs, and it approaches the target, then it positions underneath the target and starts aligning rotationally. During finalization, it then closes all fingers until it can confirm a stable grasp and enters the perch state. **b**, **c** Still images (videos in supplementary material) of the tactile perching procedure for a steeply inclined cylindrical (Trial XV) and T-Bar target (Trial XX), respectively.

This is further illustrated in Fig. 5, which shows the 3D trajectory of selected trials. E.g., in trial III the MAV makes contact immediately during the initial approach and therefore aligns quickly with the target, while in trial XI the MAV performs multiple passes of the search pattern until contact occurs and therefore aligns later. Additionally, Fig. 6 shows the *x*, *y*, and *z* position, arm opening states, contact data, and state-machine states over the full duration of Trial III. After takeoff, the MAV executes the search pattern while opening and closing its fingers. A single contact occurs at *t* = 15 s, which triggers the MAV to safely approach the target from below and perform alignment. Finally, all fingers close, sufficient contact is detected at around *t* = 35 s, and a stable grasp is confirmed, triggering the perch state.

## Discussion

In this work, we demonstrate how tactile feedback and a passively closing, underactuated gripper design can enable aerial perching to be more robust to target pose estimate uncertainties and target shapes and sizes. Drawing inspiration from nature, we show how in robotics the sense of touch can be a valuable complement to other senses, such as vision, especially when they are inhibited. The key contributions of this work are a compliant anthropomorphic hand equipped with soft tactile pads that act as sensorized phalanges, enabling an aerial tactile perching framework that continuously refines the MAV pose during the perching maneuver using embodied tactile feedback, as well as a tactile-based grasp validation strategy that ensures secure attachment before finalizing the perch. This work shows the importance of tactile sensing, providing feedback directly in the task space, i.e., the relative pose between the MAV and the target, as well as grasp stability, to achieve robustness in aerial robotic applications.

With the proposed tactile-based approach, the Monte–Carlo experiments show that the MAV can robustly perch with a success rate >99% for initial position offsets of up to 0.6 m, whereas the baseline only achieves ~0.1 m. This performance is also validated in experiments with the physical system, which also achieves successful perching with an initial offset up to 0.6 m. The trials with the physical prototype confirm the same offset robustness on both cylindrical and T-Bar targets, demonstrating the grasp versatility of the proposed gripper.

By adjusting its yaw angle during the perching maneuver, the MAV can also successfully align with rotational offsets of the target. From the Monte-Carlo simulation visualized in Fig. 3, we observe that both the feed-forward baseline and the proposed tactile-based approach achieve a success rate >99% for initial yaw offsets of at least up to 45°. This is mostly due to the compliance of the gripper, which naturally allows for some misalignment. However, using the tactile feedback, this bandwidth increases to ~60°. We also validate this behavior in the physical experiments, where the MAV successfully perches with an initial yaw offset of up to 20°.

Naturally, the maximum size of a perching target is constrained by the mechanical design of the gripper: longer phalanges allow the system to grasp larger targets. However, initial misalignment can prevent the gripper from fully enveloping the target, limiting its effective grasping capability. As shown in the right plot of Fig. 3, the tactile-based approach maintains a success rate above 99% for targets with radii up to 0.15 m, whereas the baseline method is limited to radii of ~0.1 m. For targets smaller than 0.02 m in radius, both methods fail to achieve successful perching. This failure is not due to insufficient enveloping, but rather because such small targets do not trigger enough contact sensors in the perched state to validate the grasp and register a successful perch.

In the perching applications outlined in this work, we expect the target to not always be aligned horizontally, but rather exhibit an inclination angle. In the Monte-Carlo simulations, we observe that both the feed-forward baseline and the proposed tactile-based approach successfully perch across the full range of inclination angles up to 45°, whereas the baseline method is slightly less reliable above 30°. This is due to the gripper’s compliance: as long as the target’s inclination remains within the gripper’s reach, each finger individually establishes contact with a different phalanx and adapts to the contact, slowly pulling the MAV underneath for a stable perch.

The tactile search pattern has a significant impact on the performance of the aerial tactile perching strategy. This process essentially constitutes a coverage path planning problem, where the MAV must systematically explore the region around the estimated target location to maximize the probability of contact. In particular, by increasing the area of the search pattern, the MAV can successfully find and perch on targets located further away from the initial estimate. However, it also increases the time required to complete the perching maneuver. As shown in the left plot of Fig. 3, success rates improve compared to the baseline method with larger initial offsets, but within the baseline method’s feasible range the perching time nearly doubles. The search pattern should therefore be matched to the expected uncertainty of the target estimate, minimizing perching time while maintaining a high success rate. Possible patterns include sinusoidal patterns, spirals, or raster scans, though platform dynamics must be considered, as patterns with sharp turns may be infeasible for the MAV. We select the sinusoidal pattern as the best compromise between success rate and perching time for the expected uncertainty of the target estimate.

The proposed tactile-based perching strategy has limitations and failure modes that need to be considered. The perching procedure relies on the assumptions A.1 and A.2. As previously discussed, too large initial offsets and target sizes violate these assumptions and will lead to failure of the searching procedure or the grasp, respectively. The same would apply for a violation of assumption A.3, where unexpected contacts would prevent the search procedure from converging to the target. In a fully integrated system, it would be expected that these assumptions are validated either by an operator via a tele-operation interface or by an onboard perception system. Another failure mode can occur at the boundary of the 95% success rate interval in positional offsets, i.e., when the target is located just on the edge of the search pattern. In these rare instances, the distal phalanx of a finger can get stuck above the target, i.e., preventing the MAV from moving underneath the target to execute the perching maneuver. However, this failure mode can be detected by monitoring the time series data of the control error in TOUCHED and APPROACH states, allowing the MAV to restart the approach from the abort state.

## Methods

### Manufacturing the sensorized digits

Each digit of the compliant finger assembly comprises three integrated components: a rigid structural backbone, a soft frictional interface, and an embedded tactile sensing pad. For these elements to function as a unified phalange, they must be securely bonded. In biological soft-rigid composites, strong integration is often achieved through gradual material transitions; however, such graded interfaces are difficult to reproduce in engineered systems. Instead, we employ a carefully selected combination of materials and bonding procedures that provides robust adhesion while maintaining compliance at the contact surface.

The rigid backbone of each phalanx is fabricated from PLA using fused deposition modeling (FDM) 3D printing. Each segment incorporates dedicated through-holes sized to route 22 AWG wires from the tactile sensors through the finger structure. The soft tactile pads are cast from EcoFlex-30 silicone, selected for its low Shore hardness and durability under repeated contact.

The pad geometry is designed as a semi-ellipsoid to approximate the curvature of the human fingertip and maximize contact area. A negative mold of this geometry is 3D-printed in PLA. To integrate the tactile sensor, a square copper foil patch is prepared and a 22 AWG lead wire is soldered to it. This copper-foil assembly is placed flat at the base of the mold, with the lead routed upward. This configuration ensures strong bonding between the foil and the silicone during casting while keeping the wire accessible for subsequent integration.

The silicone mixture is then poured into the mold, fully encapsulating the copper foil such that, after curing, the sensing element lies just beneath the outer surface of the pad. Once cured, the thin silicone layer covering the foil is carefully removed by light surface abrasion, exposing the sensing area without compromising the embedded structure.

To assemble the composite phalanx, the attachment surface of the PLA backbone is first sanded to increase surface roughness and promote adhesion. A flexible, silicone-based adhesive compatible with both PLA and EcoFlex is then applied. The cured tactile pad is aligned and pressed onto the phalanx, with the sensor wire routed through the corresponding pass-through hole. After adhesive curing, this process yields a durable yet compliant interface capable of withstanding repeated loading during grasping tasks. Lastly, each wire is connected to an MPR121 capacitive sensing controller located at the base of the MAV. When a phalanx contacts a conductive object, the resulting change in capacitance provides a clear indication of touch, enabling the hand to detect interactions in real time.

All design files to realize our anthropomorphic tactile hand are publicly accessible through our repository (https://github.com/BioMorphic-Intelligence-Lab/feely_drone).

### Integration with the aerial platform

Integrating the anthropomorphic tactile hand with an aerial platform requires a compact and lightweight electronics architecture. For this purpose, we use a SpeedyBee FS225 V2 5” quadrotor frame paired with a 45A BL32 4-in-1 ESC that drives four Emax ECO II Series 2207 motors. A Pixracer R15 flight controller running PX4 receives position trajectories from a RaspberryPi 5 companion computer, which also interfaces with a Teensy 4.0 responsible for controlling the three Feetec STS3032 servo motors that actuate the fingers. For position control, the MAV receives position measurements from a Motion Capture (MoCap) system as a proof of concept. It has been shown that the position estimate can also be achieved on-board the MAV using a single camera and an IMU^43^. The same companion computer processes the signals from the tactile sensors described in the following subsection. All components operate from a single 4S battery mounted on the underside of the MAV.

### Processing tactile awareness

The nominal joint positions of the three revolute joints of each robotic finger are chosen such that, in the absence of actuation, the gripper naturally rests in a fully closed configuration due to the joints’ torsional stiffness. This enables the gripper to support the full weight of the MAV when perched without any energy consumption. Each finger is actuated by a tendon routed around a spool attached to the distal phalanx, at the fingertip. Each phalanx *i* is equipped with binary contact sensors that produce a contact signal ${{\mathcal{C}}}_{i}$ upon touch.

In order to infer information about the environment from binary contact signals, the system requires knowledge of the location of the respective contact sensor. This information can be obtained by solving the steady-state dynamics of each robotic finger under a given actuation. Each robotic finger is modeled as a kinematic chain governed by the standard manipulator equations, which result from Lagrangian model analysis^44^. Therefore, the state of the *j*-th robotic finger ***ξ***_*j*_ follows:

1 $${{\bf{M}}}_{j,{\mathcal{E}}}({{\boldsymbol{\xi }}}_{j})\ddot{{{\boldsymbol{\xi }}}_{j}}+{{\bf{C}}}_{j,{\mathcal{E}}}({{\boldsymbol{\xi }}}_{j},\dot{{{\boldsymbol{\xi }}}_{j}})\dot{{{\boldsymbol{\xi }}}_{j}}+{{\bf{D}}}_{j,{\mathcal{E}}}\dot{{{\boldsymbol{\xi }}}_{j}}+{{\bf{G}}}_{j,{\mathcal{E}}}({{\boldsymbol{\xi }}}_{j}{,}^{{\mathcal{W}}}{{\mathbf{\Omega }}}_{{\mathcal{B}}})+{{\bf{K}}}_{j,{\mathcal{E}}}({{\boldsymbol{\xi }}}_{j}-{{\boldsymbol{\xi }}}_{j,0})={{\bf{A}}}_{j,{\mathcal{E}}}{\tau }_{j,{\mathcal{E}}}+{{\bf{J}}}_{j,{\mathcal{E}}}^{T}({{\boldsymbol{\xi }}}_{j}){{\bf{f}}}_{j,{\mathcal{E}}},$$

where Table 1 defines all symbols. Under the assumption of quasi steady-state movement (i.e. ${\ddot{{\boldsymbol{\xi }}}}_{j}\approx 0$ and ${\dot{{\boldsymbol{\xi }}}}_{j}\approx 0$) and no external forces (${{\bf{f}}}_{j,{\mathcal{E}}}\approx 0$) the above allows us to extract the steady state configuration ***ξ***_*j*,*ss*_ of the finger given some actuation ${\tau }_{j,{\mathcal{E}}}$ by solving

2 $${{\bf{G}}}_{j,{\mathcal{E}}}({{\boldsymbol{\xi }}}_{j}{,}^{{\mathcal{W}}}{{\mathbf{\Omega }}}_{{\mathcal{B}}})+{{\bf{K}}}_{j,{\mathcal{E}}}({{\boldsymbol{\xi }}}_{j}-{{\boldsymbol{\xi }}}_{j,0})={{\bf{A}}}_{j,{\mathcal{E}}}{\tau }_{j,{\mathcal{E}}}\,.$$

Given the non-linear nature of ${{\bf{G}}}_{j,{\mathcal{E}}}({{\boldsymbol{\xi }}}_{j}{,}^{{\mathcal{W}}}{{\mathbf{\Omega }}}_{{\mathcal{B}}})$ this equation does not have a closed form solution. However, we can solve for ***ξ***_*j*_ using a numerical approach, such as Newton’s method, where a few iterations are sufficient. For better readability, we define the function ${\bf{g}}({\boldsymbol{\xi }})\in {{\mathbb{R}}}^{3}\to {{\mathbb{R}}}^{3}$:

3 $${\bf{g}}({\boldsymbol{\xi }}):= {{\bf{G}}}_{j,{\mathcal{E}}}({{\boldsymbol{\xi }}}_{j}{,}^{{\mathcal{W}}}{{\mathbf{\Omega }}}_{{\mathcal{B}}})+{{\bf{K}}}_{j,{\mathcal{E}}}({{\boldsymbol{\xi }}}_{j}-{{\boldsymbol{\xi }}}_{j,0})-{{\bf{A}}}_{j,{\mathcal{E}}}{\tau }_{j,{\mathcal{E}}}$$

Finding the roots of **g**(***ξ***) is then equivalent to finding the steady state configuration that satisfies Eq. (2). The Newton’s Method step then takes on the form:

4 $${{\boldsymbol{\xi }}}_{j,k+1}={{\boldsymbol{\xi }}}_{j,k}-{{\bf{J}}}_{{\bf{g}}}^{-1}({{\boldsymbol{\xi }}}_{j}){\bf{g}}({{\boldsymbol{\xi }}}_{j})\,,$$

where **J**_**g**_(***ξ***_*j*_) is the Jacobian of the function with respect to the entries of ***ξ***_*j*_. In practice, a few iterations suffice to converge to the steady-state configuration ***ξ***_*j*,*ss*_. Having obtained ***ξ***_*j*,*ss*_ for each finger, we can solve the forward kinematics problem for each of the sensing pads, yielding their positions in the body frame ${\mathcal{B}}$. When any contact sensor is active, the contact location is now known, enabling the system to realign with the target by repositioning toward the contact location.

**Table 1:** Mathematical nomenclature used in this paper

Symbol	Definition
${\boldsymbol{\zeta }}={\left[{{\bf{p}}}_{{\mathcal{B}}}{{\mathbf{\Omega }}}_{{\mathcal{B}}}\right]}^{T}\in {\mathbb{SE}}(3)$	Quadrotor configuration (pose)
${{\boldsymbol{\tau }}}_{{\mathcal{B}}}\in {{\mathbb{R}}}^{4}$	Quadrotors control inputs.
${{\boldsymbol{\xi }}}_{j}={\left[{\xi }_{i,1}\ldots {\xi }_{i,3}\right]}^{T}\in {{\mathbb{R}}}^{3}$	Joint angles of the *j*-th robotic finger.
${{\bf{M}}}_{j,{\mathcal{E}}}({{\boldsymbol{\xi }}}_{j}),\,{{\bf{C}}}_{j,{\mathcal{E}}}({{\boldsymbol{\xi }}}_{j},{\dot{{\boldsymbol{\xi }}}}_{j})\in {{\mathbb{R}}}^{3\times 3}$	*j*-th robotic finger’s mass-and Coriolis matrix.
${{\bf{K}}}_{j,{\mathcal{E}}},{{\bf{D}}}_{j,{\mathcal{E}}}\in {{\mathbb{R}}}^{3\times 3}$	*j*-th robotic finger’s joint stiffness and damping matrices of the robotic finger.
${{\bf{G}}}_{j,{\mathcal{E}}}({{\boldsymbol{\xi }}}_{j}{,}^{{\mathcal{W}}}{{\mathbf{\Omega }}}_{{\mathcal{B}}})\in {{\mathbb{R}}}^{4\times 1}$	Gravity contribution to the *j*-th finger joint.
${{\bf{A}}}_{j,{\mathcal{E}}}\in {{\mathbb{R}}}^{4\times 1}$, ${\tau }_{j,{\mathcal{E}}}\in {\mathbb{R}}$	*j*-th input matrix and tendon tension
${\mathcal{B}}{{\bf{p}}}_{{\mathcal{E}}}\in {{\mathbb{R}}}^{3}$	End-Effector (EE) position in ${\mathcal{B}}$ as computed by the forward kinematics ***f***(***ξ***).
${{\bf{J}}}_{{\mathcal{E}}}({\boldsymbol{\xi }})\in {{\mathbb{R}}}^{3\times 4}$	*ξ*-dependent EE Jacobian matrix.
${{\bf{J}}}_{{\mathcal{B}},{\mathcal{E}}}({\boldsymbol{\xi }})\in {{\mathbb{R}}}^{3\times 6}$	*ξ*-dependent contact Jacobian matrix for the base.
${\mathcal{C}}=\left[{{\mathcal{C}}}_{1}\,\cdots \,{{\mathcal{C}}}_{i}\right]\in {{\mathbb{B}}}^{9}$	Vector of all binary contact signals.
${{\bf{f}}}_{{\mathcal{E}}}\in {{\mathbb{R}}}^{3}$	External force at the EE.
${{\boldsymbol{\zeta }}}_{{\mathcal{T}}}={\left[{{\bf{p}}}_{{\mathcal{T}}}{{\mathbf{\Omega }}}_{{\mathcal{T}}}\right]}^{T}\in {\mathbb{SE}}(3)$	Perching target pose

In order to obtain binary contact signals from each phalanx, we mount copper foil on each phalanx and connect it to a capacitive sensing board that measures raw capacitance. The distal phalanx of each finger carries foil on both front and back surfaces, forming a single contact interface, while the middle and proximal phalanges carry foil only on the front. This layout allows touch detection regardless of whether contact occurs inside or outside the hand’s grasp.

Contact is detected by deviations from each phalanx’s nominal capacitance: when touching a conductive object, the object becomes part of the capacitor and decreases the measured value. Thresholding this deviation yields a binary contact signal ${{\mathcal{C}}}_{i}$ for each phalanx *i*, as shown in Fig. 2.

### Rejecting contact disturbances

Intentional contact during perching can cause disturbances that may destabilize the vehicle. Instead of directly controlling interaction forces, we limit the approach velocity so that any resulting disturbance remains within the attitude controller’s rejection capability. Considering a worst-case impact, i.e., contact at maximum moment arm ${r}_{\max }$ with velocity aligned to the contact normal, the disturbance torque is conservatively estimated as

5 $${\tau }_{\max }\approx {r}_{\max }\frac{mv}{\Delta t},$$

where *m* is vehicle mass, *v* is velocity, and *Δt* is the impact duration. To stay within the controller’s maximum rejectable torque *τ*_ctrl_, we choose the command velocity of the search pattern vector field at 1.0 m/s. In practice, the compliant structure of the fingers further reduces peak impact forces, making this bound conservative.

### Search pattern selection

The search pattern strongly influences perching performance, requiring a trade-off between mean time-to-contact and robustness to large pose estimate uncertainties, a trade-off between coverage completeness and path efficiency common in coverage-path-problems^45^.

**Fig. 7:**
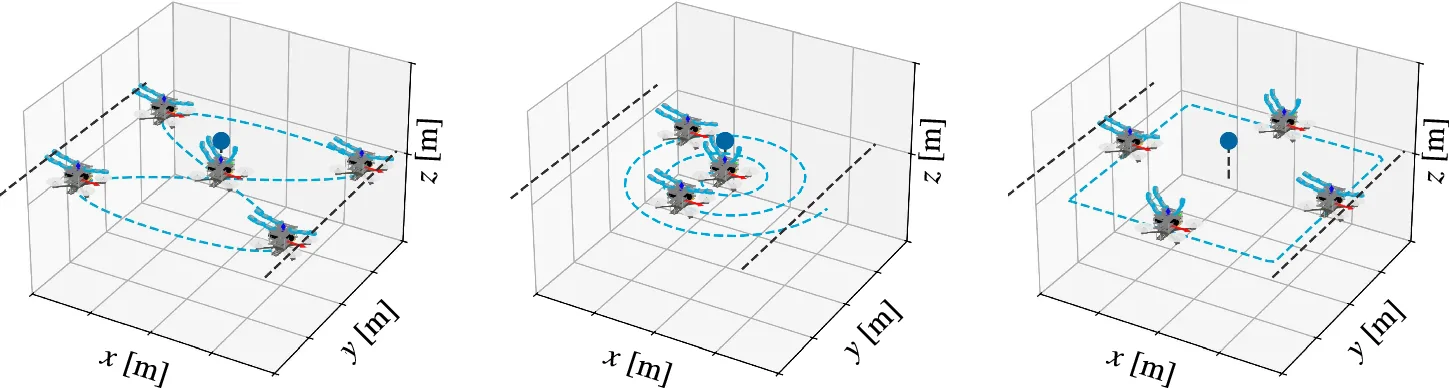
Visualization of the three search patterns considered in this work: sinusoidal, spiral, and square raster scan. The sinusoidal pattern provides a smooth, dynamically feasible trajectory approximating an optimal boustrophedonic (zigzag) sweep across the target area. The spiral pattern densely fills the search region by expanding outward in a continuous curve, while the square raster scan follows a back-and-forth path along the perimeter of the search region. These patterns each represent different trade-offs between coverage efficiency and robustness to pose uncertainty.

We consider three patterns: sinusoidal, spiral, and square raster scan visualized in Fig. 7. The problem of choosing a search pattern can be understood by casting the task as a coverage-path-problem^45^: hereby the sinusoidal pattern is a dynamically feasible, smooth approximation of the optimal boustrophedonic (zigzag) pattern for covering a rectangular cell. Compared to the square raster, it covers the interior of the search region rather than only its perimeter; compared to the spiral, which, while dense, revisits intermediate radii and thus reaches large offsets slowly, it expands outward more rapidly, which leads us to select the sinusoidal pattern as the best compromise between success rate and perching time for the expected uncertainty of the target estimate.

### Simulation environment

The simulation environment used to evaluate the proposed tactile perching strategy closely approximates the physical system while enabling rapid Monte-Carlo analysis. The simulation is implemented in the Genesis World simulator^42^, which automatically generates a dynamic model from a Unified Robot Description Format (URDF) description of both the MAV and the gripper, that includes all effects of inertia and gravity and implements a quadratic penalty formulation for enforcing rigid-body constraints. The MAV is modeled as a six-DoF system, and each finger as a three-link kinematic chain actuated by a single tendon. Additionally to the standard dynamic model we further add tendon based actuation and joint stiffness. The total additional torque ${\tau }_{i,j}^{* }$ acting on joint *j* of finger *i* is given by

6 $${\tau }_{i,j}^{* }={r}_{i,j}{f}_{i}+{K}_{i,j}({\xi }_{i,j}-{\xi }_{i,j,0})$$

where *r*_*i*,*j*_ is the distance from the tendon mounting point to the joint axis, *f*_*i*_ is the tendon tension, *K*_*i*,*j*_ is the joint stiffness (equal for all joints and measured from the real system), *ξ*_*i*,*j*_ is the joint angle, and *ξ*_*i*,*j*,0_ is the nominal joint angle for a fully closed finger. For each link the geometry is represented as a collection of primitive shapes that can make and break contact with the environment.

The simulation represents tactile sensing through the known contact forces: if a phalanx experiences a contact force above a threshold *ϵ*, the sensor outputs a binary contact signal indicating whether any portion of its geometry intersects with the environment. Low-level position, attitude, and rate control is handled by a standard cascaded controller. These controllers receive noisy measurements of position, orientation, and their corresponding velocities. Gaussian measurement noise is injected independently for each DoF to mimic the noise of a motion capture system as employed in the real system^46^. The noise terms for the *i*-th DoF, *w*_*i*_, follow:

7 $${w}_{x},\,{w}_{y},\,{w}_{z} \sim {\mathcal{N}}\left(0,\,{(0.02{\rm{m}})}^{2}\right),\quad {w}_{{\rm{rot}}} \sim {\mathcal{N}}\left(0,\,{(1.{0}^{\circ })}^{2}\right),$$

8 $${w}_{{v}_{x}},\,{w}_{{v}_{y}},\,{w}_{{v}_{z}} \sim {\mathcal{N}}\left(0,\,{(0.01{\rm{m}}/{\rm{s}})}^{2}\right),\quad {w}_{{v}_{{\rm{rot}}}} \sim {\mathcal{N}}\left(0,\,{(0.{1}^{\circ }/{\rm{s}})}^{2}\right).$$

The simulator uses a fixed-step integrator with a timestep of 0.01 s to execute the Monte-Carlo trials. For each trial, the MAV is initialized with zero attitude and placed at a uniformly sampled position on a plane at 0.25 m above the origin, spanning a 2 m × 2 m area. The full simulation implementation, including the URDF models and control pipeline, is publicly available in our repository(https://github.com/BioMorphic-Intelligence-Lab/feely_drone).

## Supplementary information

Supplementary Information

Supplementary Movie 1

Supplementary Movie 2

## Data Availability

All experiment data can be found via the project repository https://github.com/BioMorphic-Intelligence-Lab/feely_drone.

## Code availability

All generated software and design files can be found via the project repository https://github.com/BioMorphic-Intelligence-Lab/feely_drone.
