# Supplementary material for: Aerial tactile perching via an anthropomorphic hand with embodied soft tactile receptors
Source: Npj Robot. 2026 Aug 22;4(1):35. doi: 10.1038/s44182-026-00109-9 (PMC13499708; doi:10.1038/s44182-026-00109-9)
Supplement: Supplementary file 1 [file 44182_2026_109_MOESM1_ESM.pdf]

Supplementary Movie 1

Supplementary Movie 1 presents animations of the simulated experiments conducted for this study.

The movie opens with a single simulated trial for the angular offset case, in which the drone executes the search pattern highlighted in gray. Throughout all single-trial animations, the current target position estimate is shown in red, the current reference position in blue, and the commanded velocity is indicated by a yellow arrow; green circles denote the phalange positions as computed from the steady-state equations, and blue arrows represent the contact forces.

The following two trials demonstrate, respectively, a position offset on a cylindrical target and an inclination offset on an H-Bar target, both using the same visualization scheme.

The movie concludes with an animation of 100 Monte Carlo trials for a cylindrical target under a positional offset, with each drone's trajectory rendered as a blue trace.

Supplementary Movie 2

Supplementary Movie 2 presents recordings of the physical prototype experiments.

The movie opens with a close-up of a successful perch on a cylindrical target, followed by top- or side-view footage of all trials in the order of their appearance in Figure 4. The top view is used for cylindrical target trials involving position and angular offsets, while the side view is used for T-Bar target trials and targets involving inclination offsets.
